# Medical Ghost-Writing

**DOI:** 10.4103/0973-1229.33006

**Published:** 2008

**Authors:** Elise Langdon-Neuner

**Affiliations:** * *Editor-In-Chief of The Write Stuff, the journal of the European Medical Writers Association*

**Keywords:** *Ghost-writing*, *Ghost authorship*, *Author's editor*, *Acknowledgement*, *Pharamaceutical industry*, *Publication ethics*

## Abstract

Any assistance an author receives with writing a scientific article that is not acknowledged in the article is described as ghost-writing. Articles ghost-written by medical writers engaged by pharmaceutical companies who have a vested interest in the content have caused concern after scandals revealed misleading content in some articles. A key criterion of authorship in medical journals is final approval of the article submitted for publication. Authors are responsible for the content of their articles and for acknowledging any assistance they receive. Action taken by some journals and medical writer associations to encourage acknowledgement is an uphill task in the light of disinterest from the pharmaceutical industry and ignorance or similar lack of interest by those who agree to be named authors. However, acknowledgment alone is not sufficient to resolve medical ghost-writing; issues of how the acknowledgement is formulated, permission to acknowledge and access to raw data also need to be tackled.

## Introduction

Medical ghost-writing is a new term, different from the ghost-writing of autobiographies, fiction and political speeches. The ghost-writers in medicine are medical writers used by pharmaceutical companies or contract research organisations and medical communication agencies that serve the industry. Some medical writers are employed by the industry or its service agencies, others are self-employed and work under contract.

Articles written by medical writers are published in medical journals. These articles can influence doctors and policy makers in their decisions that effect health. Therefore the articles have marketing potential and there is a feeling that articles associated with manufacturers of pharmaceuticals make exaggerated promises and omit information that might disadvantage their products - but whereas advertisements carry the name of the manufacturer, a ghost-written article does not.

Medical ghost-writing has attracted the ire of the mass media and those campaigning for ethics in the dissemination of medical research information. A statement in the published article acknowledging the role of the medical writer is seen as the solution to ghost-writing. A number of guidelines aiming at transparency have been produced by organisations of journal editors, namely the International Committee of Medical Journal Editors (ICMJE, 2006; *http://www.icmje.org*; see specially ‘II.A.2. Contributors Listed in Acknowledgments’ which is related to ghost-writing) and the World Association of Medical Editors (WAME, 2005; *http://www.wame.org/wamestmt.htm#ghost*), by organisations of medical writers, namely the European Medical Writers Association (EMWA; Jacobs and Wager, 2005), and the American Medical Writers Association (AMWA; Hamilton and Royer, 2003); they have also been produced by medical writers for the pharmaceutical industry (The good publication practice: guidelines for pharmaceutical companies, GPP; Wager, 2003). Nevertheless guidelines seem to be largely ignored. I say ‘seem’ because naturally nobody knows how many articles are ghost-written.

In this article I will look at the current understanding of what constitutes medical ghost-writing, writing assistance and scientific authorship and I will critically review the extent to which acknowledgement is the solution to the problem.

## The Importance of Definition

It would be nice to start this article with a definition of ‘medical ghost-writing’. But ‘medical ghost-writing’ is an evolving term shunned by medical writers as pejorative (Jacobs and Wager, 2005) and used carelessly to embrace more than it should. Furthermore the very term itself has been blamed for the persistence of the practice because it implicitly identifies medical writers as the wrongdoers, detracting from the more serious ethical problem of the production of ghost-written articles for purposes of marketing (Moffatt and Elliott, 2007).

Authors of articles published in medical journals used to write their articles themselves. Some received writing assistance from an author's editor who edited articles for spelling and grammatical errors and improved style. This is the evolutionary stage reached by the ICMJE guidelines, which many - but not all - medical journals have adopted or have selected parts to follow. The guidelines give ‘people who provide writing assistance’ as an example of those who should be listed in the acknowledgements. Ghost-writing is not mentioned. Yet it's ‘ghost-writing’ not ‘writing assistance’, which is also still common, that has generally caused outrage in the mass media as well as in some medical journals.

Dictionaries (e.g. *Concise Oxford Dictionary, Merriam-Webster Dictionary*) define a ghost-writer as a person employed to write material for another person, who is named as author:

ghost-writer - artistic or literary hack doing the work for which his employer takes credit (Sykes, 1985: The Concise Oxford Dictionary, 1985, p416).

This person writes rather than edits. The identity of the writer does not need to be concealed to satisfy the definition. But concealment is key to the term ‘medical ghost-writing’. Therefore a medical ghost-writer is a writer whose name does not appear anywhere in a published article. In 2001, Reidenberg *et al*. proffered the definition ‘An individual who assists with the writing of a paper but does not meet all three ICMJE criteria for authorship and whose contribution is not acknowledged’. This would include both undisclosed authors' editors and medical writers. (For the three criteria, see the fourth paragraph below.)

Current attempts in the Internet to define ‘medical ghost-writing’ are along the lines, as exemplified in *Wikipedia*, that it is the practice of writers paid by pharmaceutical companies to draft articles for marketing purposes on which physicians and scientists from academia are credited with authorship to enhance the article's credibility and give the impression it is from an unbiased source (http://en.wikipedia.org/wiki/Ghostwriter#Medical). Note the shift from assistance after the author has written the draft as described above to the assistant writing the draft.

Gøtzsche *et al.* (2007) edged the term's evolution further to include unacknowledged statisticians, who they call ghost authors. In their study they compared clinical trial protocols provided by an ethics committee with corresponding publications reporting the results in medical journals. Ghost authorship existed if individuals who wrote the trial protocol, performed the statistical analysis or wrote the manuscript were neither listed as authors nor acknowledged in the manuscript. With this definition they found ghost authorship for 33 of the 44 trials they investigated.

Ghost-writing is therefore perceived nowadays as any undisclosed influence from industry, rather than academics receiving just a bit of help with their grammar and writing style. This distinction is important because author's editors, particularly those offering help to authors who are not native speakers of English, can be caught up in the ghost-writing net by the failure of the ICMJE and journal's instructions to distinguish *writing assistance* from *ghost-writing*. Although both should be transparent, there is a difference in degree.

What has remained constant throughout the evolution is the certainty that the ghost-writer is not the author. Authors belong on the title page, whereas ghost-writers are nowhere to be found on the paper, although they can be exorcised by appearance in the acknowledgements. The ICMJE guidelines (see *II.A.1. Byline Authors* at http://www.icmje.org/) consider an author to be someone who has made substantive intellectual contributions to a published study. Three criteria have to be fulfilled to warrant authorship. 1. A substantial contribution needs to have been made to the conception and design, acquisition of data or analysis and interpretation of data. 2. In addition the author must have drafted the article or revised it critically for important intellectual content. 3. Lastly, to qualify, the author must also approve the final version of the article to be published.

At workshops I have given for medical writers, which include discussion of authorship, it has become apparent that some medical writers believe they fulfil the first two criteria for authorship but are adamant that they cannot be authors because they do not approve the final version of the manuscript. This is corroborated by the medical writer Keith Dawes (2007), who mentions that medical writers might have prepared 90% of the paper but do not have the final say on the contents, which is always at the discretion of the author or sponsor. This of course begs the question of whether the sponsor is an author.

Medical writers are not alone in failing the final approval criteria. Where one author has written the article, other named authors might not have approved the final version and should not therefore be named as an author according to the ICMJE guidelines. One in five authors in a study of Cochrane reviews was found not to have been involved in drafting or revising the manuscript (Mowatt *et al.*, 2002). The ICMJE authorship criteria are not free from controversy but so far nobody has questioned the final approval criteria, although the problematic of each author guaranteeing the integrity of co-author's contributions has been discussed in the context of contributorship described later.

## The Importance of Authorship

Authorship pinpoints an accountable person. Scientists who have conducted research should be answerable for published reports of the research. Doctors advising patients and researchers conducting studies to advance scientific knowledge, rely on the integrity of published reports. Thus the anonymous medical ghost-writer who was asked in an interview with CBC news whether he/she had a bad conscience felt safe to answer, “The way I look at it, if doctors have their name on it, that's their responsibility, not mine” (*www.cbc.ca/consumersmarket/files/health/ghostwriting*).

Authors are responsible for deciding whether a ghost remains in the underworld or is brought to light in the acknowledgements. The ICMJE and WAME guidelines referred to above place responsibility on authors to identify anyone who provided writing assistance as well as to disclose the funding source for this assistance. The EMWA guidelines state that it is important for medical writers to realise that if they agree to be listed as an author they take public responsibility for research (Jacobs and Wager, 2005).

Authorship does not have the same remarkable career advancement and reputation benefit for writers as it does for academic authors, therefore it is not something medical writers will press for, even if they could. But why should author's editors/medical writers be concealed?

### Medical Writing and Concealment

Some medical journal editors believe that participation of writers paid by the pharmaceutical industry results in inevitable bias, regardless of whether the writer's role is disclosed (Griffin-Sobel, 2005). WAME, however, recommends journals make it clear that medical writers can be legitimate contributors:

To prevent some instances of ghost authorship, editors should make clear in their journal's information to authors that medical writers can be legitimate contributors and that their roles and affiliations should be described in the manuscript (WAME 2005)

Some medical journals, through their instructions to authors and editorials, have endorsed a valid role for medical writers in medical journal publication.

An intent to deceive is not generally the reason for employing a medical writer. Medical writers are employed to process a heavy workload that would be a waste of researcher resources. They collate data from large multi-centre studies and write up the results for licence applications to regulatory authorities. They are also used to write publications based on these results. Their expertise is in compiling data and presenting it in tables, graphs and text. They also have experience in writing manuscripts for which scientists rarely receive training during their academic education. Accordingly the main reasons to use medical writers are efficiency and speed. But their use has gone beyond publications associated with the results of clinical trials to include review articles and opinion pieces and it is this that has particularly attracted concern.

Most medical writers are themselves PhDs, which enables them to understand the science, but few have been trained in linguistics. Author's editors are more likely to be linguists. Healy and Chattel (2003) in the study detailed below believed the use of medical writers is likely to result in more research entering the public domain (and it is generally thought faster), than if writing is left to busy investigators. They also hoped that some medical writers might encourage disclosure of conflicts of interest. It's a fair assumption that because the focus of medical writers' work is writing and publication they are generally more aware than scientists and doctors of the rules of publication ethics through familiarity with guidelines. No studies have shown whether the increased involvement of medical writers over recent years has resulted in the writing style in articles becoming more erudite.

I suspect there are four reasons for non-disclosure of writing assistance/writing:

1. The first is that authors are embarrassed to admit to having received assistance. This is more likely to apply to authors using author's editors. Some editors feel a paper should reflect authors' writing skills rather than their ability to choose a good writer/editor. They ask how authors, who are too busy or whose writing skills are so poor that they need help, can guarantee the quality of their research. There is however more sympathy if English is not the author's first language (*http://www.wame.org/wame-listserve-discussions/ethical-guidelines-plagiarism-and-ghost-writing*).
2. The second reason is ignorance of the need to disclose. Many researchers (50 from 66 questioned) at one university faculty were unaware of the ICJME authorship criteria and 62% disagreed with the criteria (Bhopal *et al.*, 1997).
3. The third is a concern that journals are less willing to accept the article for publication (Wilde, 2005). This concern is legitimate (Griffin-Sobel, 2005). But the chances of rejection are greater if a medical writer's involvement is not disclosed but suspected or discovered. Among others, the *American Journal of Medicine* has said that they reject papers, which have too much company-speak (Larkin, 1999). The statement ‘supported by an unrestricted educational grant’ in a paper may be interpreted as ‘ghost-written’.
4. The fourth reason for non-disclosure is to stealthily market drugs. Healy and Cattell (2003) summed up the scenario of articles perceived as written by opinion-leaders with minimal company representation and non-declaration of other non-academic inputs increasing the likelihood that the articles will be influential with prescribers and purchasers. We know that readers are sceptical of articles with declared pharmaceutical industry involvement (Chaudhry *et al.*, 2002).

## Examples of Ghost-writing in the Literature

Secrecy is always suspect. Studies and commentaries suggest 11-50% of studies are ghost-written (Flanigan *et al.*, 1998; Healy, 2004; Wilde, 2005; Woolley *et al.*, 2006). The few reported incidents come to light through uncommon events: a whistleblower, a court order to produce documents or an academic who refuses an invitation to be named as author. An editor or peer reviewer cannot normally be expected to identify ghost-written papers, although routine checks of the File Properties of electronically submitted MS Word manuscripts can reveal the document's author and drug company involvement (Madsen, 2005; Ebell at *http://www.wame.org/wame-listserve-discussions/ethical-guidelines-plagiarism-and-ghost-writing.* Accessed 2 May 2007). Reported examples of ghost-writing have covered up problems with drugs, sought to circumvent the Federal Drug Agency's prohibition on advertising off-label indications and endeavoured to create a market for a drug.

Some ghost-writers have spoken out about instructions they have received on the slant and emphasis to be used in articles (Wilde, 2005). Marilynn Larkin (1999) was given an outline, references and a list of drug-company-approved phrases. Ronni Sandroff was told what to play up and what to play down (Larkin, 1999). Susanna Dogson was asked to slant a paper in favour of the drug company (Wilde, 2005). Susanna Rees (2003) was told to replace the names of drug company employees with those of the named authors in the File Properties of MS Word manuscripts.

David Franklin became concerned about patient safety and spoke out against his employers Parke-Davis (Lenzer, 2003). Doctors were encouraged through articles published in medical journals to prescribe Neurontin, a drug licensed for treating epilepsy, for a wide range of off-label disorders. Off-label sales accounted for 78% of the drug's sales. Franklin sought legal advice for fear that his employers might retaliate against him for raising questions about the legality of the ghost-writing schemes used to promote off-label sales of the drug (ibid).

Documents disclosed in court proceedings allowed Healy and Cattell (2003) to conduct a study on ghost-writing and its effectiveness. They investigated two types of articles with sertraline in the title. Sertraline is the generic name for Pfizer's antidepressant drug Zoloft. One type of article was listed on a document requisitioned during court proceedings from a medical communications agency contracted by Pfizer. Some articles on the list were complete except for the name of the author, which was noted as ‘TBD’ or ‘to be determined’. Of the 85 articles on the list, 55 were written by medical writers employed by the agency. The remaining 30 were funded by Pfizer or used economic models based on data provided by Pfizer, where the authors would not have had access to the raw data. Only two articles declared writing assistance. All the articles reported positive results. The second type of article was not on the list but found by searching Medline and EMBASE. Of these 41 articles, 18 reported positive, 3 ambiguous and 20 negative findings. The articles on the agency's list that had been published appeared in more prestigious journals than the independent articles indicating the effectiveness of the agency's work and influence of the credentials of the named authors in increasing the chances of acceptance for publication.

Documents disclosed in court proceedings also revealed that Merck hired ghost-writers to write articles favourable to its painkilling drug Vioxx (generic name rofecoxib) (Krumholz *et al.*, 2007). Vioxx entered the market in 1999 but was voluntarily withdrawn in September 2004 because it increased cardiovascular disease. In-house scientists at Merck had from the beginning suspected that the drug increased thrombus formation. Nevertheless outside academic authors of a study the company had sponsored changed a manuscript at Merck's request to hedge their findings. One named author of a Vioxx article subsequently said he had never seen any revision of the paper (Barnett, 2003) and another that he had had little to do with the research (Liane, 2005).

David Healy also provided an example of the third type of event that has exposed ghost-writers (Select Committee on Health, 2004). He was sent a review article that had been written by a ghost-writer. He was asked to approve the article and put his name to it as the author. He found the claims made for the drug exaggerated and sent back his own version. The pharmaceutical company that had sent the article to him preferred their original version, which they said, had important commercial points in it that were missing from Healy's own version. The pharmaceutical company's original version was subsequently published in a journal with the name of another scientist as author. David Healy's disclosure of the name of this author alerted me to make enquiries when the journal where I worked as managing editor received an article from the author.

## The Attitude Towards Who Writes the First Draft

If a person employed by a commercial enterprise or organisation with an agenda to promote writes the first draft, the potential for bias is increased. But readers have a chance to weigh potential bias in the balance if full information about the document's history and financing is disclosed and if the raw data of original research is available for scrutiny.

As long ago as 1998, David Sharp, former editor of the *Lancet*, stated that assistance with translation or through an author's editor service of the sort that many universities offer, is entirely legitimate (Sharp, 1998). In contrast, he considered the practice of a medical writer producing a first draft in which the writer's employer, rather than the named author, has first sight and input should be outlawed. These sentiments were echoed more recently by Special Assistant US Attorney Cathy Young Thomer who considers ghost-writing to be a fraud when the writing precedes the author (Jirik, 2000).

There is some ambivalence among medical writers over writing of the first draft. The GPP and medical writer association ethics guidelines focus on guidance for medical writers in preparing drafts of documents publishing the results of clinical trials in journals. The need for close consultation between medical writers and authors is emphasised. The guidelines state that authors must determine the content of the articles and the contribution of the writer should be acknowledged. The GPP consider it inappropriate for medical writers to prepare the first drafts of editorials or opinion pieces. The background commentary to the EMWA guidelines warns that medical writers may qualify for authorship of review articles if they conduct an extensive literature search. These guidelines therefore envisage medical writers preparing review articles, which can be as influential as editorials (GPP in 2003, EMWA in 2005).

## Problems with Acknowledgement

Although medical writers are not ghost-writers if they are acknowledged, acknowledgement alone does not satisfy current concerns. There are three aspects of acknowledgement that need to be considered. One is the information that should be included. The second is the requirement, adopted by some journals, in the ICMJE guidelines that authors obtain permission to acknowledge from all those mentioned in the acknowledgements. The third is whether the authors had access to raw data.

Journals differ widely in the amount of guidance their instructions to authors give on acknowledgements, from giving no guidance – one could suspect that they care little about the subject themselves – to just referring to the ICMJE guidelines or variously requiring authors to give the name, the role and funding source of any person providing writing assistance.

The EMWA guidelines suggest wording that has been endorsed by some journals (e.g. *Arthritis Research and Therapy*, a BioMed Central journal) in their instructions to authors: ‘We thank Jane Doe who provided medical writing services on behalf of XYZ Pharmaceuticals Ltd.’

This formula, however, is not so detailed as the one used in a review article in *Neuropsychopharmacology* (Nemeroff *et al.*, 2006), which was strongly criticised by *Science* in its news section (Holden, 2006): ‘We thank Sally Laden for editorial support in developing early drafts of this manuscript. We maintained complete control over the direction and content of the paper. Preparation of this report was supported by an unrestricted grant from Cyberonics, Inc.’ The objection was that it did not include an explicit statement that the medical writer, Sally Laden, was paid by Cyberonics. In the context of the failure of the authors, one of whom was the editor-in-chief of the journal, to disclose their conflicts of interest, this critique is somewhat disproportionate (Shashok, 2007), but it does highlight the need for unambiguous statements in the acknowledgements about who paid the medical writer and what ‘unrestricted’ really means.

Krumholz *et al.* (2007) in an article in which they ask, what we have learnt from Vioxx, suggest the wording: ‘Representative from *XYZ* drafted the manuscript; the authors were responsible for critical revisions of the manuscript for important intellectual content.’ This suggestion also lacks an explicit statement about the medical writer's source of funding.

Clearly, a cohesive approach is called for. It would be helpful if guidance could come from a central source, e.g. the ICMJE or WAME. I drew the ICMJE's attention to this need and submitted proposals for consideration at their meeting in April last year that their guidelines on acknowledgement should be amended to require the following information:

- The name of the person who provided writing assistance
- The name of the person/company/organisation who paid for the writing assistance
- The name of the person who wrote the first draft
- The names of the people who approved the final draft
- If a named author prepared the first draft, the name and funding source of the person who provided writing assistance, if any.

The names of the people who approved the final draft could, for example, include the publication manager in a drug company or communication agency and would be in line with WAME's statement:

…responsibility for ghost written manuscripts goes beyond individual authors. Other parties, including companies-such as marketing, communications and medical education companies who are paid to assist pharmaceutical and medical device companies in disseminating favourable messages about their products – may initiate the sequence of events for which the author is the final and most easily identified participant. These other participants are also responsible for ghost written manuscripts and addressing their roles should be part of the solution (WAME, 2005).

The ICMJE published their updated guidelines in October 2007. Disappointingly they had made no changes to their acknowledgement requirements.

Some journals, for instance the *Lancet*, insist on a signed statement from the person giving permission to be named in the acknowledgements section (*http://www.thelancet.com/authors/lancet/authorinfo*). This is not without its difficulties. There maybe legitimate reasons why a medical writer does not wish to be acknowledged, not the least being the fact that authors have made changes to the final version that the writer considers inappropriate. These might just be language changes that would reflect badly on writing skills of a professional editor/writer to whom they would be credited. But they may also relate to changes that are unacceptable on ethical grounds. Acknowledgement might also be complicated by the paper passing through several stages with different writers/editors involved. The position on acknowledgement when these problems arise is not clear. The EMWA guidelines recognise that medical writers have a right to withdraw their names in exceptional circumstances but are silent about whether the medical writers should inform the journal of their involvement and subsequent withdrawal (Jacobs and Wager, 2005).

In the reporting of clinical trial results, the problem of ghost-writing goes beyond concealment of the writer and failure of the named authors to approve the final version of the article. Even authors who approve the final version might not have had access to the raw data but only tables compiled from raw data. They maybe familiar with the raw data generated by their own centre but not that from all other centres spread through several countries. David Healy (2004) regards articles that result from the ghost-writing process which do not offer a fair representation of the underlying data as the problem rather than ghost-writing itself. Gøtzsche *et al.* (2007) and others have pushed for protocols to be published in the belief that the only way to ensure honesty is access to raw data. Pharmaceutical companies consider raw data proprietary and strongly resist all moves to make their data available for public scrutiny. The current move is to force publication of documents that accompany applications to licensing authorities for drug licences. Either the applicants or the authorities would make these documents available upon the grant of the licence. But even more needs to be done towards access to raw data.

### Action to Ensure Acknowledgement

Guidelines increase awareness. An EMWA-AMWA members' survey found an association between members' familiarity with guidelines and more frequent acknowledgement of medical writers (Hamilton and Jacobs, 2006). This indicates that medical writers encourage authors to disclose their involvement according to the guidelines. Medical writers have also published articles promoting ethical practices (e.g. Dawes, 2007; Woolley, 2006). But guidelines can be a foil, with all those whose writing had never sought to deceive complying, while business carries on as usual for the dishonest sector. Furthermore, the willingness to comply with guidelines must go beyond that of the medical writer. Employers need to be persuaded. Currently there is little incentive for pharmaceutical companies to stop the practice of ghost-writing.

The shame about the well-meant GPP guidelines designed for company sponsors of large clinical trials is that they have been endorsed by only a few pharmaceutical companies even though employees from some other companies helped to develop them. Furthermore one communications company that agreed to recommend the guidelines to its clients has subsequently been involved in a ghost-writing story (Fugh-Berman, 2005). This hints that medical writers, who work hard through their medical writers associations (EMWA in Europe, AMWA in American, AMWA in Australia and a similar organisation that is being created in India) to promote acknowledgement, would themselves be happy to be relieved of their underworld status but their employers prefer to keep them there. Indeed in an EMWA-AMWA members' survey only 3% of respondents were opposed to acknowledgment (presented by Adam Jacobs at the EMWA conference in Lyon, 2006; unpublished).

Journals can ask the right questions, which few are doing at the moment. One of the few is the *BMJ* which questions authors of review articles and editorials about whether they have written the article themselves or it has mainly been written by a medical writer (*http://resources.bmj.com/bmj/authors/article-submission/Submitting-an-article-to-the-BMJ*). Journals can also have an antenna for ghost-writing signs and of course avoid being tempted themselves by the lure of industry patronage.

There has been a proposal to change authorship to contributorship (Rennie, 1997). By this system the specific contribution made by every person associated with the publication is listed. This should reveal who wrote the paper. Unfortunately, although the system is recommended by the ICMJE, it has only been implemented by some large journals.

Authors get more than an honorarium from selling their name. They accumulate articles on their CVs without effort, gaining career advancement and a reputation as an opinion leader, which in turn attracts lavish invitations – all expenses paid – to speak at congresses in exotic locations. A suggestion has been made to name and shame authors who plagiarise (Chalmers, 2006), which could be extended to authors who fail to acknowledge writers. But editors might be reluctant to do this. There seems to be a protectionist attitude towards authors who are members of the same community as journal editors, a fear that innocent scientists or physicians may have their careers ruined or an anxiety about defending a libel action. The WAME policy statement advises editors who discover ghost-written articles to report the incident to the authors' academic institutions and publish a notice that the article was ghost-written along with the names of the responsible companies and submitting authors:

When editors detect ghost written manuscripts, their actions should involve both the submitting authors and commercial participants if they are involved. Several actions are possible:

1. publish a notice that a manuscript has been ghost written, along with the names of the responsible companies and the submitting author;
2. alert the authors' academic institutions, identifying the commercial companies;
3. provide specific names if contacted by the popular media or government organizations; and
4. share their experiences on the WAME Listserve and within other forums. (WAME, 2005)

The statement was prompted by a WAME Listserve discussion initiated by the editor of the *Journal of General Internal Medicine* when Adriane Fugh-Berman reported that a manuscript she had been sent by the journal to review was identical to the one she had previously been asked to author (Fugh-Berman, 2005). The name of the replacement author was never published on the Listserve or elsewhere. Hence other journal editors have been deprived of the opportunity of caution with this author, who would seem to have little to fear.

In a recent article Moffatt and Elliott (2007) ask whether the ethical problems presented by ghost-written articles would be solved if medical writers were acknowledged as authors. To be acknowledged as authors, medical writers would of course need to satisfy the current ICMJE authorship provisions as stated above. However, Moffatt and Elliott believe that what really needs to be tackled is the creditability endowed on articles by sham “named authors” from academia. They suggest that university institutions police their faculty members. I fear this is likely to be met with issues similar to those mentioned above when asking journal editors to do the task, but more weight might be added by their further suggestion of also placing responsibility with a government institution (e.g. the Office of Research Integrity). Less certain is that their suggestion of joining ghost-writers and authors in actions taken against pharmaceutical companies (such as Motus *vs* Pfizer) would act as a great deterrent. Such actions are relatively rare and execution for payment of damages and costs is likely to be pursued against the more financially viable defendants.

## Conclusions

1. Acknowledgement is necessary to exorcise ghosts.
2. Clear guidance needs to be given on the form of the acknowledgement and an answer should be found for when medical writers are not prepared to give permission for their acknowledgement because unacceptable changes have subsequently been made to their work.
3. All parties concerned need to actively support transparency: authors, journals, medical writers and the pharmaceutical industry.
4. The popular press has an important role in raising end consumer and political momentum. Patient groups that will grow with the increase in average age of Western populations could wield more influence in demanding ethics in the dissemination of medical research information.
5. Acknowledgement is not sufficient where authors do not have access to raw data.

## Questions That This Paper Raises

1. Do medical writers have a legitimate task to perform in compiling data and bringing research to publication?
2. Should the ICMJE's ‘approval of the final draft’ criterion of authorship be treated separately from the names of those who approved the final version listed in the published article?
3. Should accountability be strictly confined to those who genuinely had access to raw data and must they always be authors?
4. Should doctors whose prime job and duty is towards patients accept payment/writing assistance from pharmaceutical companies whose sole duty is to make a profit for their shareholders?
5. Is ghost-writing merely a beacon of the commercialisation of medicine against which patients have no voice?

## Issues for Further Deliberation

1. The interconnections between authors, journals and the pharmaceutical industry, a major financer of research, are very strong. Added to which the pharmaceutical industry has a powerful political lobby, especially is the USA. The people who are disadvantaged by unethical dissemination of scientific information are patients and the taxpayer - all of us.
2. Medical writers, perhaps as a proxy for their employers, rather than academic and physician authors have been the focus of attack for ghost-writing but medical writers do have a task to perform compiling data and bringing research to publication. Their associations encourage ethical practices in this task. Whether medical writers should be preparing the first drafts of opinion pieces needs careful thought.
3. Consideration might be given to transferring ‘approval of the final draft’ from a criterion of authorship to a separate list of the names of those who approved the final version published with the manuscript. Authors could still be defined as those who made substantial intellectual as well as research contributions to the work.
4. Authors can only be accountable for the raw data they have had access to. Either raw data needs to be made available for public scrutiny or those who do have access to the raw data must be made personally accountable.
5. Ghost-writing is a side product of the commercialisation of medicine. Medical writers are earning a living. Pharmaceutical companies have a duty to their shareholders to make a profit and some doctors and scientists are taking advantage of a windfall. Others have commendably resisted the temptation.

## About the Author

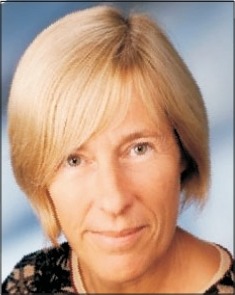



Elise Langdon-Neuner BSc has a background in science, law and biomedical publication. She was a litigation partner in a London firm of solicitors for a number of years. After moving to Austria she became managing editor of Diabetologia, the largest European diabetes medical journal. In 2004 she launched a new Elsevier journal and managed the journal through its teething years. Currently she is a senior manager at Baxter BioScience in Vienna, where her work includes editing manuscripts prepared by in-house research scientists. She is editor in chief of The Write Stuff, the journal of the European Medical Writers Association and an editorial board member of European Scientific Editing, the journal of the European Association of Medical Editors. She regularly writes articles, conducts workshops and lectures on various aspects of manuscript preparation and biomedical journal editorial policies. Her main interests are ethics in scientific publication and the future of scientific communication.
